# scGAL: unmask tumor clonal substructure by jointly analyzing independent single-cell copy number and scRNA-seq data

**DOI:** 10.1186/s12864-024-10319-w

**Published:** 2024-04-22

**Authors:** Ruixiang Li, Fangyuan Shi, Lijuan Song, Zhenhua Yu

**Affiliations:** 1 School of Information Engineering, Ningxia University, Yinchuan, 750021 China; 2 Collaborative Innovation Center for Ningxia Big Data and Artificial Intelligence Co-founded by Ningxia Municipality and Ministry of Education, Ningxia University, Yinchuan, 750021 China

**Keywords:** Generative adversarial network, Autoencoder, Single-cell sequencing, Intra-tumor heterogeneity

## Abstract

**Background:**

Accurately deciphering clonal copy number substructure can provide insights into the evolutionary mechanism of cancer, and clustering single-cell copy number profiles has become an effective means to unmask intra-tumor heterogeneity (ITH). However, copy numbers inferred from single-cell DNA sequencing (scDNA-seq) data are error-prone due to technically confounding factors such as amplification bias and allele-dropout, and this makes it difficult to precisely identify the ITH.

**Results:**

We introduce a hybrid model called scGAL to infer clonal copy number substructure. It combines an autoencoder with a generative adversarial network to jointly analyze independent single-cell copy number profiles and gene expression data from same cell line. Under an adversarial learning framework, scGAL exploits complementary information from gene expression data to relieve the effects of noise in copy number data, and learns latent representations of scDNA-seq cells for accurate inference of the ITH. Evaluation results on three real cancer datasets suggest scGAL is able to accurately infer clonal architecture and surpasses other similar methods. In addition, assessment of scGAL on various simulated datasets demonstrates its high robustness against the changes of data size and distribution. scGAL can be accessed at: https://github.com/zhyu-lab/scgal.

**Conclusions:**

Joint analysis of independent single-cell copy number and gene expression data from a same cell line can effectively exploit complementary information from individual omics, and thus gives more refined indication of clonal copy number substructure.

## Background

### Intra-tumor heterogeneity

The evolution of cancer is motivated by accumulation of various genomic mutations [1], and reconstructing tumor phylogeny allows us to better understand tumorigenesis and development [2]. In addition, tumors often consist of groups of cells with different genotypes, and these different subpopulations are known as tumor subclones that correlate with each other via a phylogenetic tree [3]. It is known that human tumors can exhibit striking intra-tumor heterogeneity (ITH) in different features such as histology, gene expression and genotype [4]. For instance, the appearance of each tumor subclone is often characterized by changes in DNA copy number as well as gene expression levels [5, 6]. ITH is one of the critical factors contributing to tumor treatment resistance and relapse due to treatment failure [4, 7, 8]. Without a clear elucidation of the ITH, clinical treatment tends to target only the primary clone, but after treatment, minor subclones may gain a growth advantage and cause the cancer to recur [9, 10]. Therefore, accurately deciphering ITH is important for understanding cancer progression and developing personalized treatment strategies.

### Single-cell sequencing

While bulk sequencing technology is a powerful means to study tumor growth processes, the average measures obtained from a large number of cells often obscure low-prevalence subpopulations or cellular states that may be useful for disease biology. Single-cell sequencing (SCS) enables a finer dissection of the cellular states of cancer [11]. The main advantage of SCS is that genomic mutations can be profiled at single-cell resolution, and this is particularly useful to accurately unmask the ITH [12]. In recent years, the rapid development of SCS technology has made it possible to simultaneously obtain multiple omics data from single cells [13–15], which enables more precise investigation of ITH and multi-molecular co-regulatory mechanisms within tissues. Specifically, integrated analysis of tumor single-cell copy number and gene expression data promises better quantification of ITH [16].

Although single-cell sequencing technology can provide high-resolution single-cell data, technical issues such as allele-dropout and amplification bias complicate the ITH analysis [17]. For instance, amplification bias may cause some genomic regions to be unevenly amplified, and this probably makes downstream copy number analysis tools miscalculate the copy numbers; ultralow sequencing coverage (typically lower than 1X coverage) makes most of the regions not being covered by sequencing reads, and such high sparsity property contributes to errors in copy number calling; allele dropout may cause a heterozygous site to be identified as homozygous state, and such biased measures of allele frequency may affect the copy number estimation accuracy when considering both read counts and allele frequency [18]. In addition, tumors are often characterized by aneuploidy, and most of the existing single-cell copy number calling methods are less effective in coping with aneuploid samples [19]. As a result, the inferred single-cell copy number profiles tend to be error-prone, and this poses a critical challenge for accurately deciphering clonal copy number substructure.

### Related studies

To date, a number of deep learning methods [19–23] have been proposed for inferring cell subpopulations from single-cell single-omics data such as single-cell DNA sequencing (scDNA-seq) or single-cell RNA sequencing (scRNA-seq) data. These methods are typically built based on autoencoder (AE) models. For instance, Dhaka [20] uses a variational autoencoder (VAE) to reveal ITH from single-cell copy number alteration (CNA) or gene expression data. bmVAE [21] clusters single-cell mutation data based on a VAE model and estimates subclonal genotypes using a Gibbs sampling method. To jointly infer tumor subclones and single-cell CNAs, rcCAE [19] employs a convolutional AE to enhance the quality of scDNA-seq data and simultaneously learn representations of cells. scDSC [22] recognizes cell identities from sparse scRNA-seq data by utilizing a hybrid model equipped with a zero-inflated negative binomial-based AE and a graph neural network (GNN). For better representation of scRNA-seq data, scDCCA [23] employs a denoising AE with a dual contrast learning module to extract valuable features for clustering cells. Considering the difference between different omics data, existing methods for clustering scRNA-seq data may be less effective in identifying tumor clones from single-cell copy number data.

To better cluster cells by exploiting more information from single cells, a number of methods [24–30] have been proposed for analyzing single-cell multi-omics data, e.g. scRNA-seq, single-cell assay for transposase-accessible chromatin using sequencing (scATAC-seq), DNA methylation measurements, and cellular indexing of transcriptomes and epitopes by sequencing (CITE-seq). For instance, coupleCoC [24] performs coupled co-clustering of single-cell multi-omics data through unsupervised transfer learning. Specifically, both cellular and genomic features are clustered simultaneously, and this can reduce noise in single-cell data and facilitate knowledge transfer between single-cell datasets. DEMOC [25] is a deep embedded multi-omics clustering method, it jointly clusters CITE-seq data by considering characteristics of transcriptome and proteome data. scMOC [26] reasons cell clusters using common measurements from matched scRNA-seq and scATAC-seq data. scMCs [27] co-models single-cell transcriptome and epigenetic data to get omics-specific and consistent representations, and fuse them into a common embedded representation. In addition, MSCLRL [30] is able to automatically learn latent representations from multi-omics data for cancer subtyping.

There are a few methods that specifically tackle the cellular correlation between DNA copy number and gene expression data [31–33]. For instance, clonealign [31] assigns scRNA-seq cells to clones inferred from scDNA-seq data. It identifies clone-specific dysregulated biological pathways that cannot be found from either individual omics alone. However, clonealign does not explicitly integrate scDNA-seq and scRNA-seq data into a single framework, thus cannot effectively exploit the complementary information from multi-omics to better explain ITH. CCNMF [32] employs a non-negative matrix factorization method to collaboratively infer tumor clones from matched scDNA-seq and scRNA-seq data. Despite that these conventional statistical methods provide valuable insights on how to infer ITH based on single-cell multi-omics data, their performance may still be limited due to the sparse and high-dimensional nature of the data. Deep learning methods for integrating single-cell copy number and gene expression data are therefore sorely required to deliver more precise indication of ITH.

### Proposed method scGAL

**Fig. 1:**
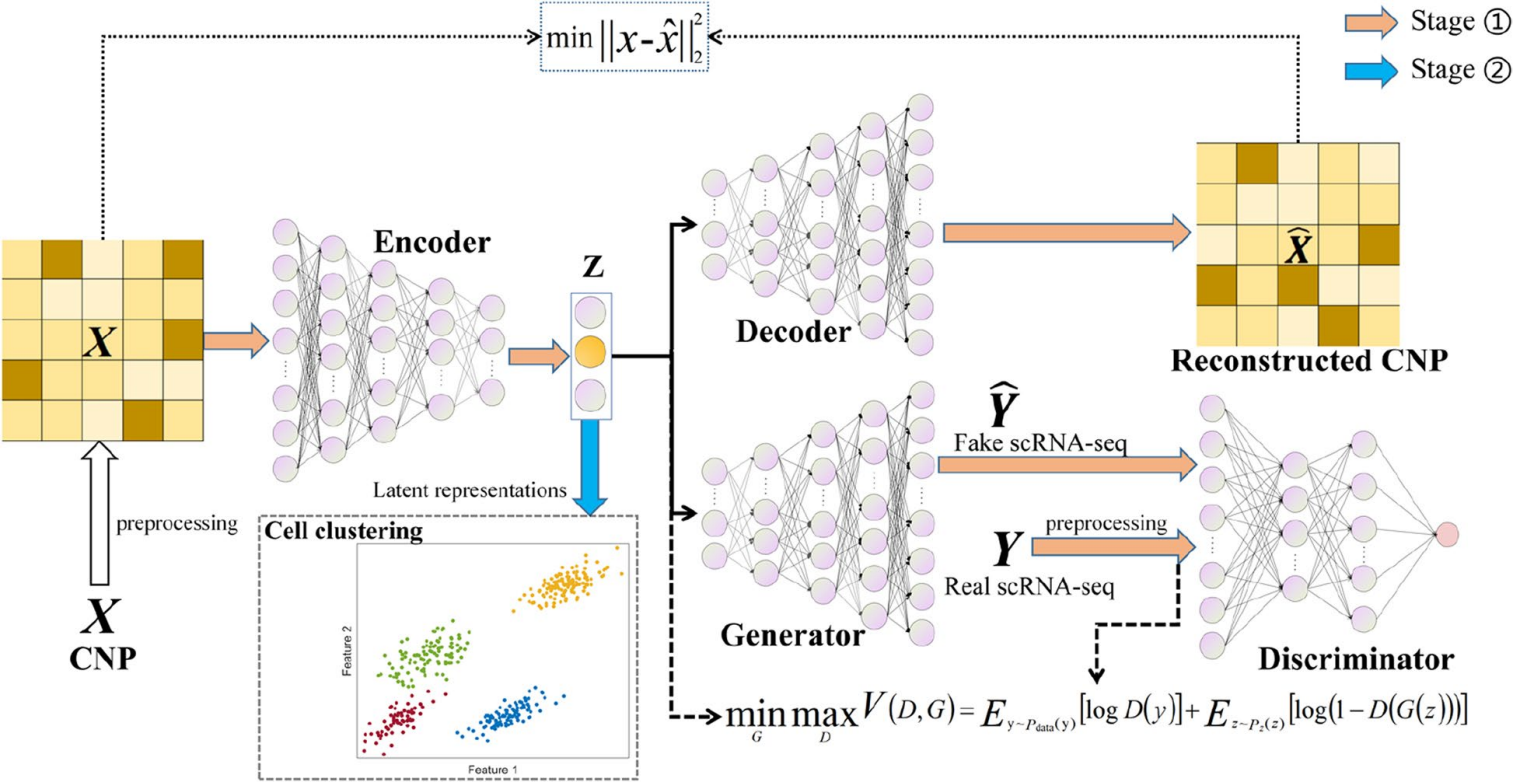
The workflow of scGAL. scGAL aims to identify clonal copy number substructure from single-cell copy number data, by borrowing complementary information from independent scRNA-seq data of the same cell line. An autoencoder (AE) is employed to learn the latent representations of scDNA-seq cells, and a generative adversarial network (GAN) is used to mimic the distribution of real scRNA-seq data given the representations. The unsupervised representation learning with combined usage of AE and GAN enables effective reconstruction of the underlying intercorrelations between single-cell copy number and gene expression data, thus making it possible to get more refined clonal copy number substructure. Based on the learned latent representations, a Gaussian mixture model is then used to identify the cell subpopulations. CNP: copy number profile

We introduce a new method scGAL for clustering single-cell copy number profiles by integrated analysis of independent scRNA-seq data from the same cell line under a Generative Adversarial Learning framework, as shown in Fig. 1. Based on the correlation between copy number and gene expression [31], this hybrid model architecture allows us to obtain semantic features for clustering single-cell copy number data, by utilizing the single-cell expression data to provide complementary information for ITH inference. This correlation can be explained from tumor evolution perspective: as tumor evolves through accumulation of genomic mutations, and the evolutionary history of tumor can be characterized by a phylogenetic tree, where distinct tumor subclones correlate with each other by sharing common mutations [34], implying that some copy number alterations can exist in multiple tumor subclones, and genes in these regions may show similar gene expressions across the subclones. Based on the common features shared by tumor subclones and the correlation between copy number and gene expression, we utilize the complementary information from gene expression data to aid in ITH inference from single-cell copy number data under an adversarial learning framework, and this results in fused low-dimensional representations of the cells for identifying tumor subclones by clustering.

scGAL first learns latent representations of scDNA-seq cells under an unsupervised manner, and then clusters the cells based on the latent representations. Specifically, it uses the encoder to project the single-cell copy number data into a latent representation, the decoder to reconstruct the copy number data and the generator to yield synthetic scRNA-seq data based on the representation. With an adversarial learning strategy to mimic the distribution of real scRNA-seq data, the complementary information from scRNA-seq data is integrated into the latent representation, and this enables a more refined indication of the subclonal architecture. The integrated analysis of scDNA-seq and scRNA-seq data also relieves the effect of technical noises present in copy number data on ITH inference. Given the learned representations, scGAL employs a Gaussian mixture model (GMM) to aggregate cells into different subpopulations.

We conduct a comprehensive evaluation of scGAL’effectiveness and robustness on real as well as simulated datasets, and compare scGAL to several similar approaches. The results demonstrate our method is able to accurately infer clonal copy number substructure and surpasses other methods.

## Results

### The workflow of scGAL

The workflow of scGAL is depicted in Fig. 1, and there are two main components that form the framework of scGAL: an AE to learn latent representation of cells from single-cell copy number data, and a GAN to embed complementary information from independent scRNA-seq data into the representation. We employ several preprocessing steps to filter and improve the copy number and gene expression data before downstream analysis. The AE takes the pre-processed single-cell copy number data as input, and reconstructs the input from a learned latent representation. The GAN module is introduced to mimic the distribution of real scRNA-seq data given the representation. We conduct joint optimization of the two network components to get semantic representations of the scDNA-seq cells, and then employ a GMM in combination with Bayesian information criterion (BIC) to identify different cell subpopulations. A hyperparameter $\lambda$ is introduced to balance the reconstruction loss of the AE and adversarial loss of the GAN when optimizing the network weights. Details regarding pre-processing of scDNA-seq and scRNA-seq data as well as model training can be found in Methods section.

For implementation of the networks, we use fully connected layers to process the data. Formally, the encoder contains four intermediate layers each with 512, 256, 128 and 64 nodes, the decoder and generator have a mirrored structure of the encoder, the latent dimension is set to 3, and the discriminator has two intermediate layers each with 32 and 64 nodes. We apply LeakyReLU activation function to all layers except the last layer of the encoder, and sigmoid activation function to the last layer of the discriminator. We also apply a Batch Normalization (BN) layer after each middle layer of the encoder and decoder to improve the training stability and generalization ability of the model. The weights are initialized using the approach introduced in [35] to make the model training faster, and also solve vanishing/exploding gradient problem. Note that the dimension size of the latent layer is an important hyper-parameter of the model, and as suggested by the results on simulated and real datasets used in this study, setting latent dimension to 3 is sufficient to obtain effective representations for clustering the cells. Detailed descriptions about the scGAL framework and implementation can be found in the Methods section.

### Evaluation on real datasets

#### Datasets

Three unpaired real datasets including a primary triple-negative breast cancer dataset (SA501) [13], a high-grade serous carcinoma (HGSC) dataset [31] and a gastric cancer dataset (NCI-N87) [16] are used to evaluate the performance of scGAL. We select these datasets for evaluation since they all contain unpaired single-cell copy number and gene expression data from the same cancer cell line, and our method can be employed to cluster the single-cell copy number data by borrowing complementary information from corresponding scRNA-seq data.

#### Selected methods for performance evaluation

We compare scGAL to two most relevant baseline methods including Dhaka [20] and RobustClone [36] that can directly cluster single-cell copy number data, as well as three scRNA-seq based methods including Seurat [37], scBGEDA [38] and scTAG [39], which are evaluated on single-cell copy number data to check if they can conquer the inherent difference between two individual omics data and accurately identify clonal copy number substructure. Furthermore, to validate the effectiveness of the GAN module employed in scGAL for integrating standalone scRNA-seq data, an AE model that has the same structure as the AE module of scGAL and only analyzes single-cell copy number data, is used as a baseline method. To evaluate the clustering performance of the methods, we employ several widely used performance metrics, including the adjusted rand index (ARI) [40] and normalized mutual information (NMI) [41] that are calculated based on ground truth labels, the silhouette coefficient [42] and Calinski-Harabasz index [43] that are used when real labels are not available.

For scGAL, we empirically determine the learning rate within a range and manually adjust it to observe its convergence during training. Furthermore, since our model takes inputs from both scDNA-seq and scRNA-seq datasets, we set the batch size of both data according to the ratio between the sizes of two datasets. We test different batch sizes (32, 64, 128), and select the one that results in fast and stable convergence of the model. Other hyperparameters are also determined through numerous trials and based on experimental results. For Dhaka, we test different learning rates and activation functions to select the best hyper-parameters. Our results suggest learning rate of 0.00001 or 0.0002 and activation function of Rectified Linear Unit (ReLU) result in better convergence of the model and higher clustering accuracy, therefore we use these hyper-parameter values to run Dhaka 20 epochs on SA501 and NCI-N87 datasets while 30 epochs on HGSC dataset. We use default parameters to run RobustClone on three datasets, and follow its pipeline of first recovering the genotype matrix and then clustering the cells. For Seurat, we conduct experiments at various values of the resolution parameter and select the optimal value based on the experimental results. As a result, we use a resolution of 0.1 on the SA501 and HGSC datasets, while a resolution of 0.2 for the NCI-N87 dataset. As a predefined number of clusters needs to be specified for scBGEDA and scTAG, we set it to the true number of clusters on each dataset, and use default values of other parameters to run scBGEDA and scTAG.

### Results on the triple-negative breast cancer dataset

We apply scGAL to the SA501 dataset that is derived from primary triple-negative breast cancer xenografts, it contains unpaired copy number and gene expression data of single cells from the same cell line. A previous study [13] has shown this dataset contains three subclones, where subclone A consists of 214 cells, subclone B contains 28 cells, and subclone C is comprised of 18 cells. The copy number differences between subclones A and B/C are mainly observed on the X chromosome, while the copy number differences between subclones B and C are less pronounced, with only significant differences observed on chromosome 11.

**Fig. 2:**
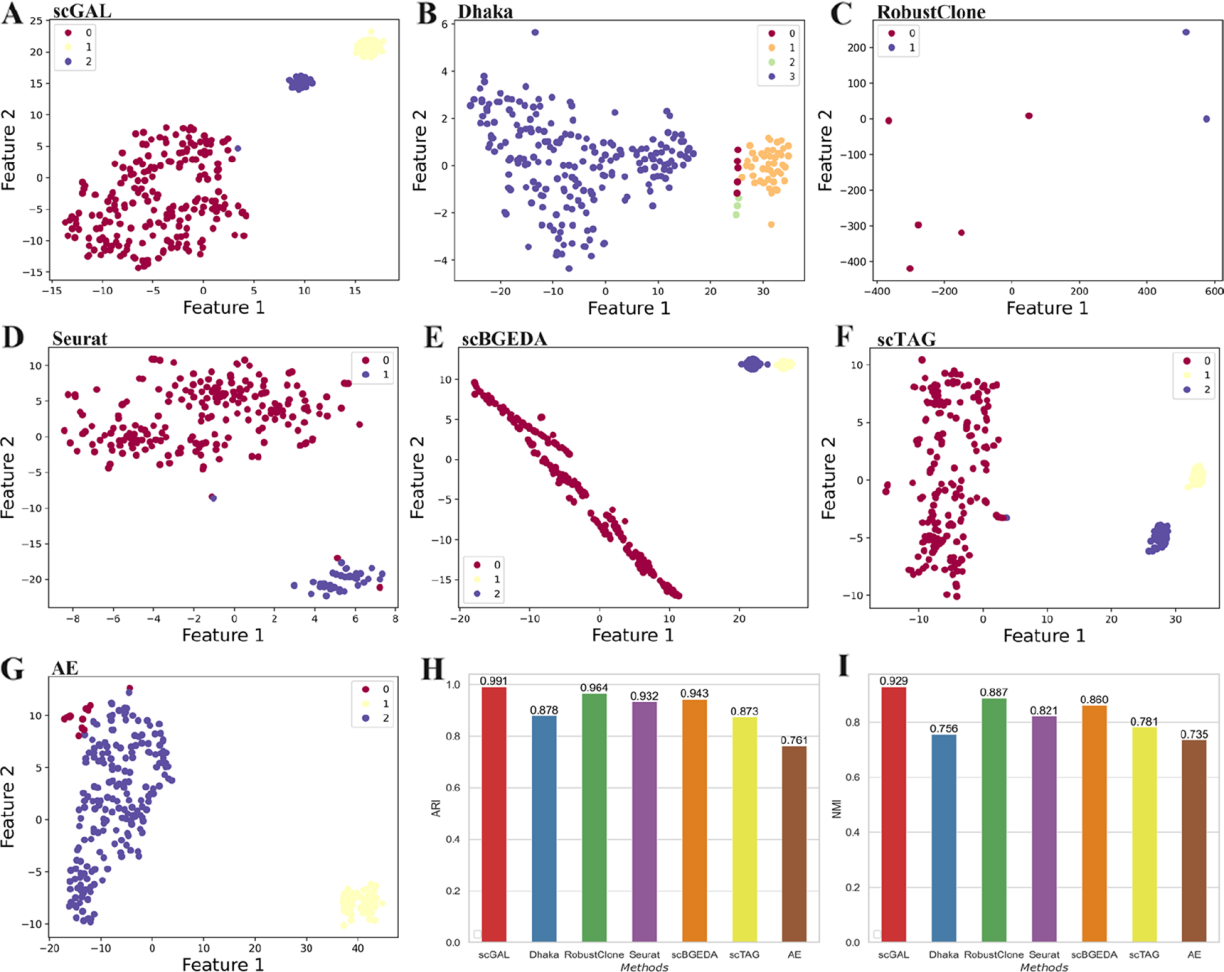
The clustering results and corresponding performance metric values of scGAL, Dhaka, RobustClone, Seurat, scBGEDA, scTAG and AE on the SA501 dataset. The AE model is used as a baseline model that only analyzes single-cell copy number data. The 2-d plots are generated by using t-SNE to project the raw data (recovered genotype matrix is used for RobustClone) or low-dimensional latent representations generated by deep learning methods into a 2D space. **A** and **G** show the results of scGAL and AE, respectively, where both scGAL and AE identify 3 subpopulations, whereas scGAL is able to better distinguish the subclones. **B**-**F** Clustering results of other methods. All of the existing methods misestimate the number of cell subpopulations except for scBGEDA and scTAG (the ground truth number of clusters is given as part of the input of scBGEDA and scTAG). It should be noted that the genotype matrix recovered by RobustClone shows many cells have the same genotype, resulting in sporadic points on the 2-d plot. **H**, **I** ARI and NMI scores of all methods

**Fig. 3:**
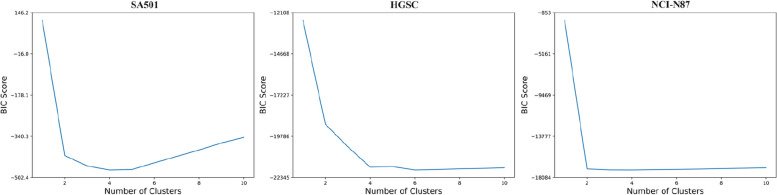
Plots of BIC score with respect to the number of clusters. Solutions containing small clusters with less than 3 cells are marked as invalid. From the valid solutions, we determine the optimal number of clusters by selecting the model with the smallest BIC score

We remove copy number data of X and Y chromosomes to attenuate the difference between copy number profiles of subclones A and B/C, then investigate if our method can still distinguish between different subclones by borrowing the complementary information from single-cell gene expression data. During the training process, we use DNA copy number matrix containing 260 cells and 19,219 genomic bins, and gene expression data of 2470 cells over 32,738 genes from the same cell line to train our model 180 epochs with a learning rate of 0.0008. The results in Fig. 2A, H and I show scGAL divides scDNA-seq cells into three subgroups with an ARI score of 0.991 as well as an NMI score of 0.929, and yields similar clustering results as previously reported. Figure 3 shows the BIC score against the number of clusters, and the clustering result with 3 clusters is selected as the best solution on the SA501 dataset.

**Fig. 4:**
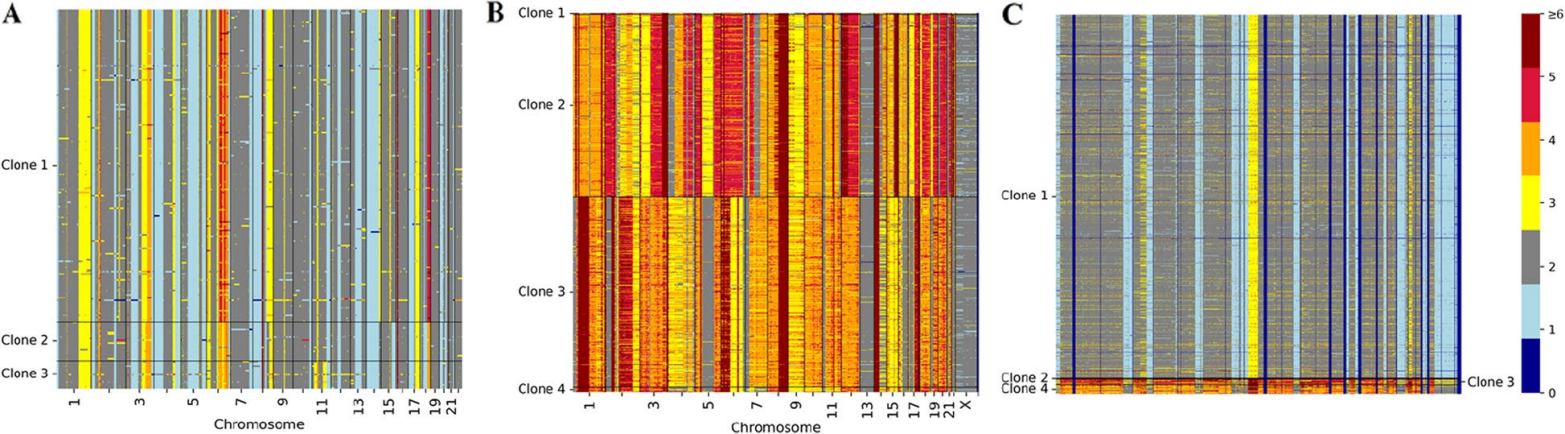
Copy number heatmaps of the SA501, HGSC and NCI-N87 datasets. Copy numbers of cells in fix-sized genomic bins are compared between distinct cell subpopulations (rows correspond to the cells and columns refer to the genomic bins). The colors represent different copy numbers. **A** Copy number heatmap of SA501 shows there are many small-sized regions across the chromosomes that have different copy numbers in subpopulation 1 compared to subpopulations 2 and 3. The differences between subpopulations 2 and 3 are mainly observed on chromosome 11. **B** Copy numbers of OV2295R (clones 1–2) and TOV2295R (clones 3–4) cell lines are significantly different, and there are also minor differences in copy numbers between cell subpopulations of the same cell line. **C** Copy number heatmap of NCI-N87 dataset. There are significant differences between the copy number profiles of distinct subpopulations. Chromosome information of the bins is not available for this dataset

**Fig. 5:**
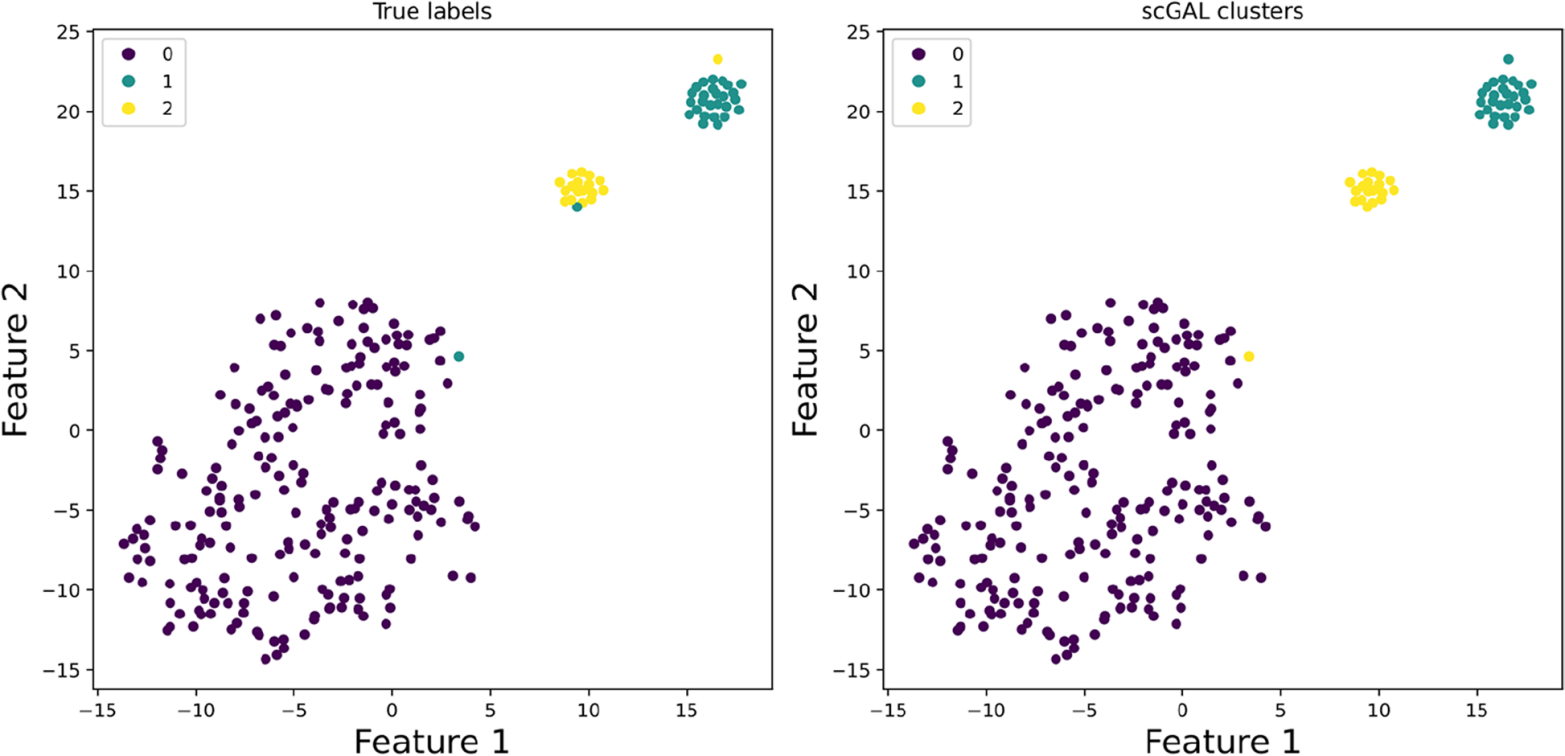
Visualization of ground truth clusters and predicted clusters of scGAL on the SA501 dataset

Although the differences between subclones A and B/C are deliberately suppressed by removing the copy number data of X and Y chromosomes, scGAL still correctly infers the underlying copy number clones by effectively integrating clonal architecture information from independent scRNA-seq data, which indicates intercorrelations between DNA copy number and gene expression data could be exploited to identify copy number clones that show less distinguishability in copy number profiles. The copy number profiles depicted in Fig. 4A show the differences between tumor clones, which further verifies the accuracy of our clustering. In addition, by comparing ground truth clusters and predicted clusters of scGAL (Fig. 5), we find that our method yields highly consistent cell labels with the ground truth, and is able to accurately identify both major and minor clusters.

We also analyze the clustering results and calculate the corresponding performance metrics for Dhaka, RobustClone, Seurat, scBGEDA and scTAG on this dataset. As shown in Fig. 2, these methods are less accurate than scGAL in clustering the cells and achieve ARI scores of 0.878, 0.964, 0.932, 0.943 and 0.873, respectively. scGAL also yields higher NMI score than other methods. In addition, the numbers of subpopulations inferred by these methods except for scBGEDA and scTAG differ from the ground truth. It is noted that the ground truth number of clusters is given as part of the input for scBGEDA and scTAG. The results also indicate integrated analysis of single-cell copy number and gene expression data under a GAN framework yields more accurate inference of tumor subpopulations, with higher ARI (0.991 vs. 0.761) and NMI (0.929 vs. 0.735) scores than the AE that uses only single-cell copy number data.

### Results on the high-grade serous carcinoma dataset

The HGSC dataset comes from two clonally related high-grade plasmacytoid carcinoma cell lines [31], which are derived from ascites (OV2295R) and solid tumors (TOV2295R) of the same patient. The scRNA-seq dataset describes gene expressions of 1717 OV2295R cells and 4918 TOV2295R cells over 32,738 genes. The unpaired scDNA-seq dataset is comprised of 371 OV2295R cells as well as 394 TOV2295R cells, and contains copy number data of 5397 genomic bins. Campbell et al. [31] construct a single-cell phylogeny using a latent tree model on copy number profiles, and the inferred phylogenetic tree contains four distinct clades. We apply scGAL on this dataset to check if our method can decipher the underlying copy number clonal structure.

**Fig. 6:**
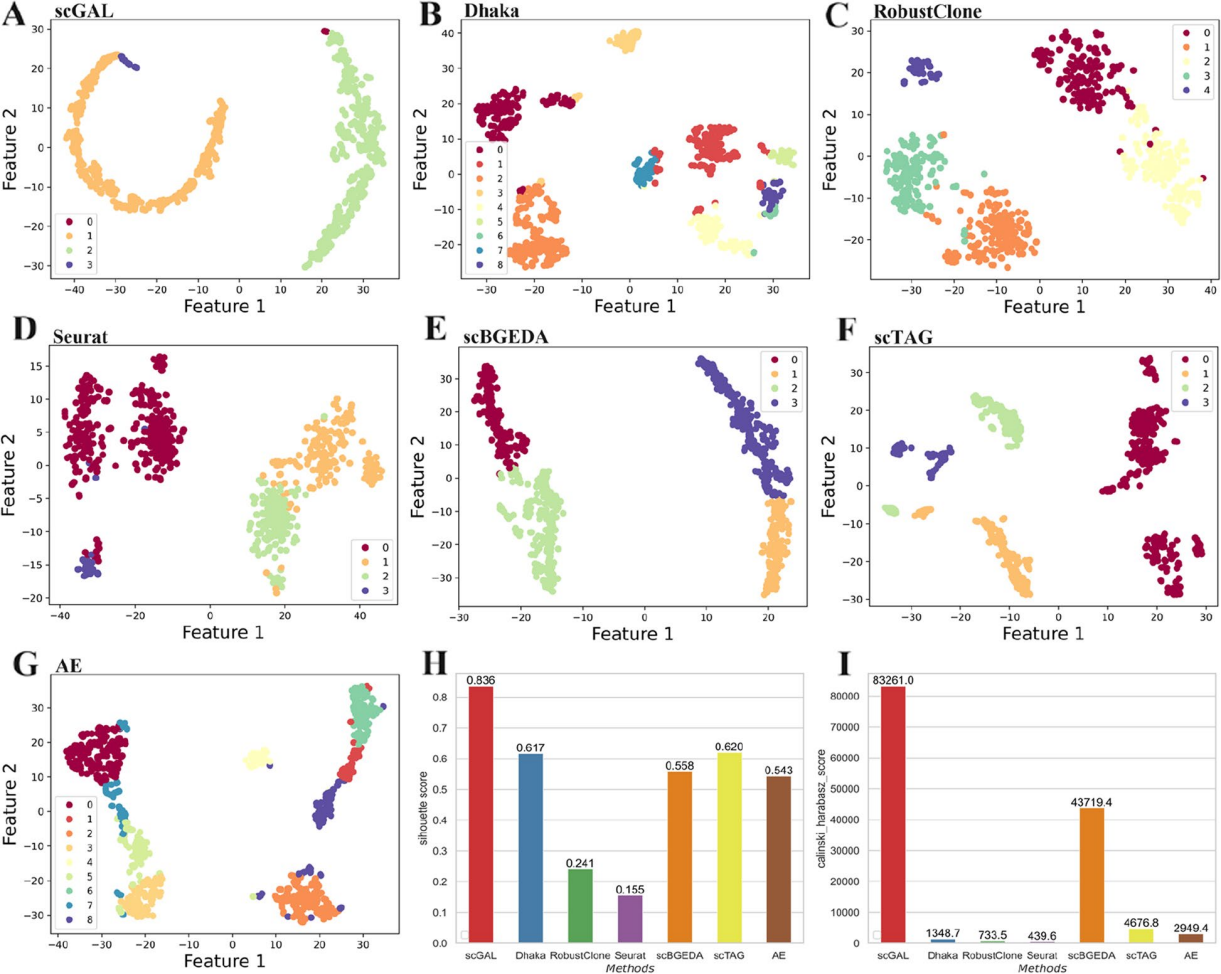
The clustering results of scGAL, Dhaka, RobustClone, Seurat, scBGEDA, scTAG and AE on the HGSC dataset. **A** and **G** show the results of scGAL and AE, respectively, where scGAL accurately identifies 4 subclones, and the AE clusters the cells into 9 subclones. **B**-**F** Clustering results of other methods. **H**, **I** Silhouette coefficients and Calinski-Harabasz scores of all methods

We train the scGAL model 120 epochs with a learning rate of 0.007, and the results in Fig. 6A show scGAL clusters the cells into four subgroups (labelled as 1–4). The BIC scores under different numbers of clusters are depicted in Fig. 3. The results are consistent with the reported results of a previous study [31] that has also clustered the scDNA-seq cells into four subpopulations. Our method successfully distinguishes the cells of TOV2295R from the cells of OV2295R, and divides the cells into two parts that are far apart from each other in the latent space. Each cell line is divided into two subpopulations, namely TOV2295R_A (384 cells), TOV2295R_B (10 cells), OV2295R_A (368 cells), and OV2295R_B (3 cells). As ground truth labels of the cells are not available, we calculate silhouette coefficient and Calinski-Harabasz score to indicate clustering accuracy. Our method achieves a silhouette coefficient of 0.836 and a Calinski-Harabasz score of 83,261. Figure 4B shows the copy number profiles of each subpopulation inferred by scGAL, and significant differences are observed between the subclones. For instance, subpopulation 1 and subpopulation 2 of the OV2295R cell line differ significantly in their copy number profiles on many chromosomes (e.g. chr 1–7,12,15–22) from subpopulation 3 and subpopulation 4 of the TOV2295R cell line. In addition, subpopulations from the same cell line show small changes in copy numbers across the chromosomes. For instance, there are slight differences in copy numbers between subpopulations 3 and 4 on chromosomes 4, 7, 10, and 14. These results suggest scGAL successfully deciphers the underlying clonal copy number substructure on the HGSC dataset.

By comparing scGAL with other methods (as shown in Fig. 6), we find that scGAL achieves the best results. The silhouette coefficients and Calinski-Harabasz scores of other methods are not competitive compared to scGAL. For instance, Dhaka and scBGEDA have silhouette coefficients of 0.617 and 0.62, respectively, while RobustClone, Seurat and scTAG have silhouette coefficients lower than 0.6. Importantly, our hybrid model significantly surpasses the baseline AE that only uses copy number data to infer subclones, which demonstrates scGAL effectively exploits complementary information from gene expression to improve inference of clonal copy number substructure.

### Results on the gastric cancer dataset

**Fig. 7:**
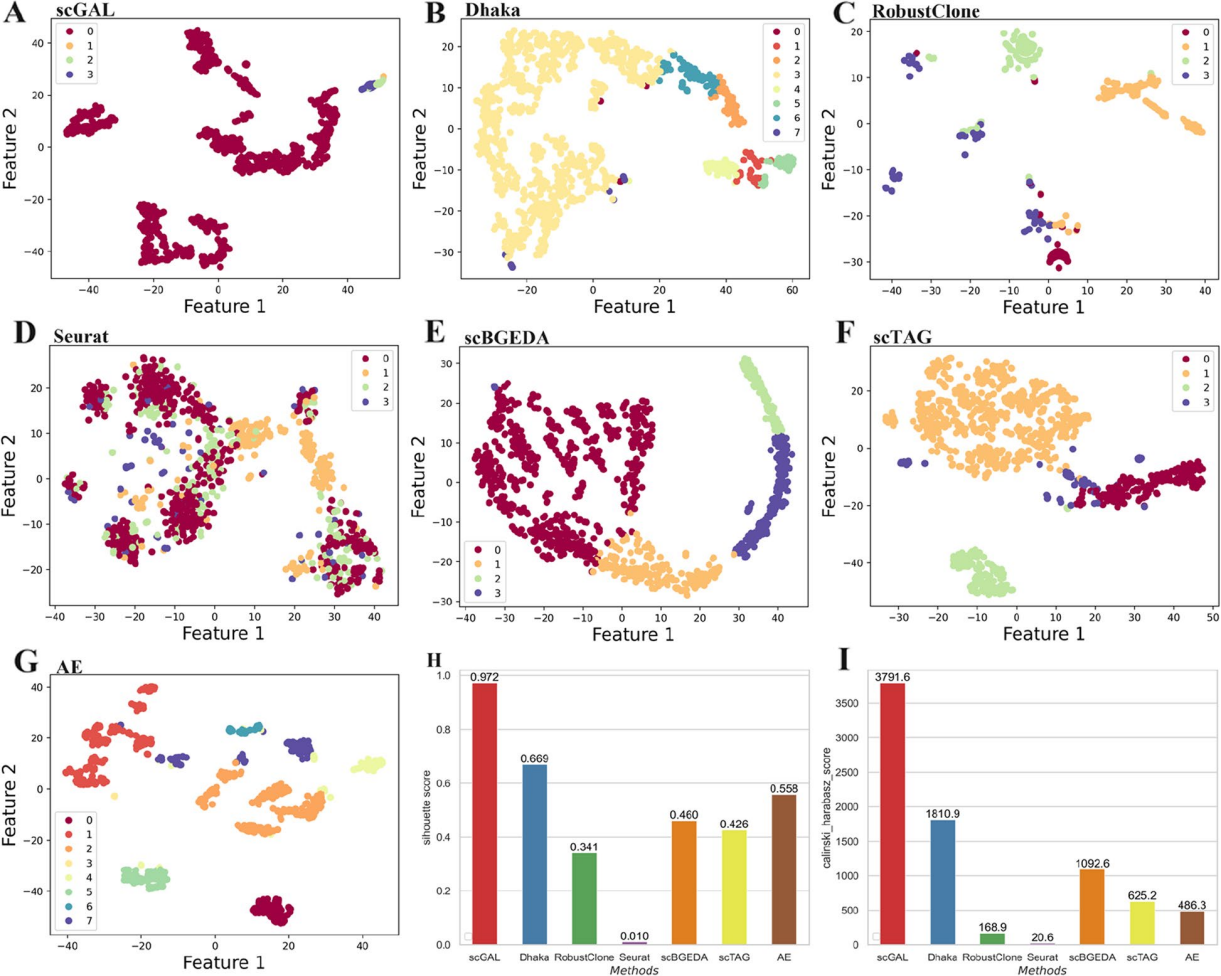
The clustering results of scGAL, Dhaka, RobustClone, Seurat, scBGEDA, scTAG and AE on the NCI-N87 dataset. **A** and **G** exhibit the results of scGAL and AE, respectively. scGAL accurately identifies 4 subpopulations, while the AE groups the cells into 8 clusters. **B**-**F** Clustering results of other methods. **H**, **I** Silhouette coefficients and Calinski-Harabasz scores of all methods

To further evaluate the effectiveness of our method, we analyze the NCI-N87 gastric cancer cell line [16], a large dataset consisting of 3246 scRNA-seq cells with 13,513 genes and 1005 scDNA-seq cells with 154,423 genomic bins. The alignments between two omics cells are unknown. Previously, Andor et al. [16] has identified four subclones by analyzing the scDNA-seq and scRNA-seq data. To assess if our method could yield similar results, we train scGAL 200 epochs with a learning rate of 0.006 on this dataset. The BIC scores under different numbers of clusters are shown in Fig. 3. The results in Fig. 7A, H and I indicate that scGAL also clusters the cells into four subpopulations with a silhouette coefficient of 0.972 and a Calinski-Harabasz score of 3791.6. These subpopulations consist of 964, 3, 14 and 24 cells, respectively. In addition, to further validate the clustering results of our method, we compare copy number profiles of the inferred subpopulations, and results in Fig. 4C show scGAL successfully assigns cells with similar copy numbers to the same cluster.

Compared to other methods (as shown in Fig. 7), scGAL exhibits the highest silhouette coefficient and Calinski-Harabasz score. Among the comparative methods, Dhaka shows relatively higher performance than others, while Seurat yields notably low silhouette coefficient and Calinski-Harabasz score, which may result from the high dimensionality of the single-cell copy number data. Similarly, the results indicate the complementary information contained in gene expression data can aid in better characterization of the tumor subclonal architecture.

### The effects of hyper-parameters

**Fig. 8:**
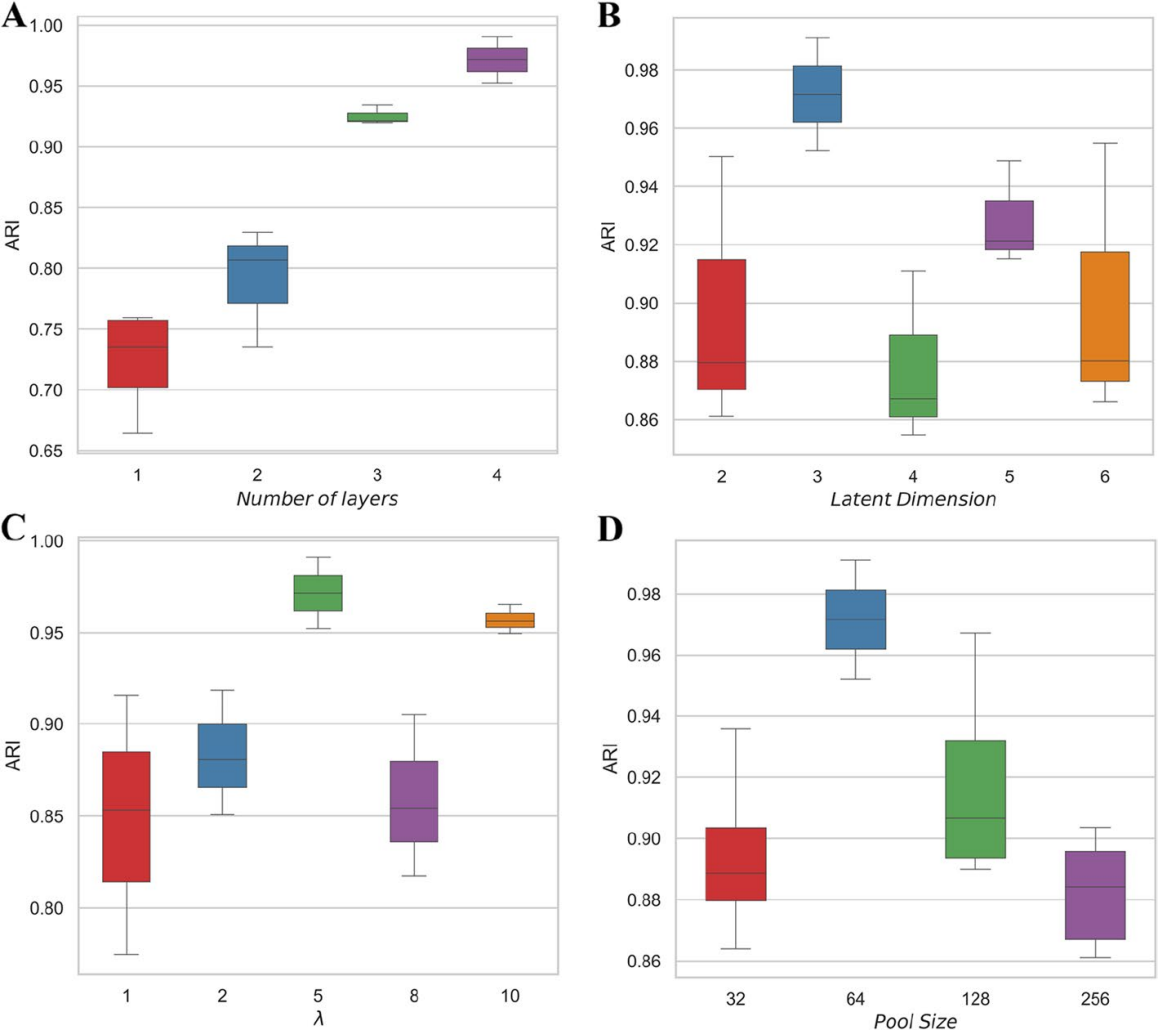
The effects of hyper-parameters on clustering performance of scGAL. For each hyper-parameter, the evaluation is conducted three times on the SA501 dataset by using different seeds. **A** Numbers of hidden layers in {1, 2, 3, 4} are tested for comparing different network architectures. The results suggest the network architecture with 4 hidden layers delivers the best results. **B** Different latent dimensions in {2, 3, 4, 5, 6} are compared, and the results indicate the best performance is obtained with latent dimension of 3. **C** Through evaluation of the effect of the hyper-parameter $\lambda$ that balances the reconstruction loss of the AE and adversarial loss of the GAN, it is found that setting $\lambda$ to 5 gives the best clustering results. **D** The size of buffer pool used to train the GAN has significant effect on model training, and buffer size of 64 is appropriate to get better clustering results

We assess the effects of network architecture related hyperparameters, such as the number of hidden layers and latent dimension, on clustering results of scGAL. As the encoder, decoder and generator networks have the same number of hidden layers, we compare the results obtained with different numbers of hidden layers in {1, 2, 3, 4}, and the corresponding sizes of the layers are set to 512, 256, 128 and 64, respectively. The evaluation results on SA501 dataset (as shown in Fig. 8A) suggest more complex network structures tend to yield more accurate clustering results, and the network architecture with 4 hidden layers delivers the highest ARI scores (median ARI is 0.972). In addition, we also investigate the effect of latent dimension on clustering performance by comparing the results based on the latent dimensions in {2, 3, 4, 5, 6}. The results in Fig. 8B indicate latent dimension of 3 generally yields better results than other settings, and increasing the latent dimension does not enhance the clustering performance.

We also evaluate the effect of hyperparameter $\lambda$ that acts as a weight factor to balance reconstruction loss of the AE and adversarial loss of the GAN (more details in the Methods section). Specifically, values in {1, 2, 5, 8, 10} are tested for $\lambda$. The results in Fig. 8C showcase the hyperparameter $\lambda$ has a significant effect on cell clustering, and the best ARI scores are obtained when setting $\lambda$ to 5. Although the optimal value of $\lambda$ may be dataset-dependent, we do not explore it on other datasets and simply use the same value of 5 on all experiments.

When optimizing the GAN module in scGAL, we use a buffer pool to store previously generated samples and use them to update the network weights (more details in the Methods section). The size of the buffer pool may have a considerable effect on optimization of the model, and therefore we test different buffer sizes in {32, 64, 128, 256} to compare the clustering results. As depicted in Fig. 8D, buffer size of 64 results in the best ARI scores, and larger buffer sizes do not yield improved clustering accuracy, which implies moderate number of generated samples should be used to train the GAN for mimicking the statistic distribution of real scRNA-seq data, thus delivering clonal structure that aligns well with the ground truth.

### Evaluation on simulated datasets

#### Simulated datasets

To comprehensively verify if the clustering accuracy can be improved when additionally incorporating independent scRNA-seq data into the analysis, we apply scGAL on various simulated datasets. As no simulation tools are currently available for simultaneously simulating single-cell multi-omics data, we are unable to simulate scDNA-seq and scRNA-seq data of the same cell line. Nevertheless, it is feasible to simulate scRNA-seq data and meanwhile use the real scDNA-seq data to construct the simulation datasets. Specifically, we combine the real scDNA-seq data of SA501 with simulated scRNA-seq data to constitute the two-omics datasets. The simulated scRNA-seq datasets are produced using Splatter software [44]. We use Splatter to estimate simulation related parameters from the real scRNA-seq data of SA501, and these parameters include data distributions, gene expression levels, inherent heterogeneity and variability. The Splatter tool allows us to capture the essential features of real scRNA-seq data and use them to generate simulated data that retains important biological properties, thus provides a reliable simulation dataset for the evaluation of our method.

There are two important parameters in Splatter package including the number of cells and probability of genes being differentially expressed (i.e., de.prob), that control the generation of scRNA-seq data. To fully assess the robustness of scGAL against changes in data size and distribution, we generate simulated scRNA-seq data under different numbers of cells and values of the de.prob. Specifically, the number of cells ranges from 1500 to 5000, and the value of de.prob changes from 0.1 to 0.4. The parameters are set by calling setParams function of the Splatter tool. These simulated datasets could provide a comprehensive evaluation of scGAL’ability to decipher ITH from single-cell copy number and gene expression data.

#### Evaluation results

**Fig. 9:**
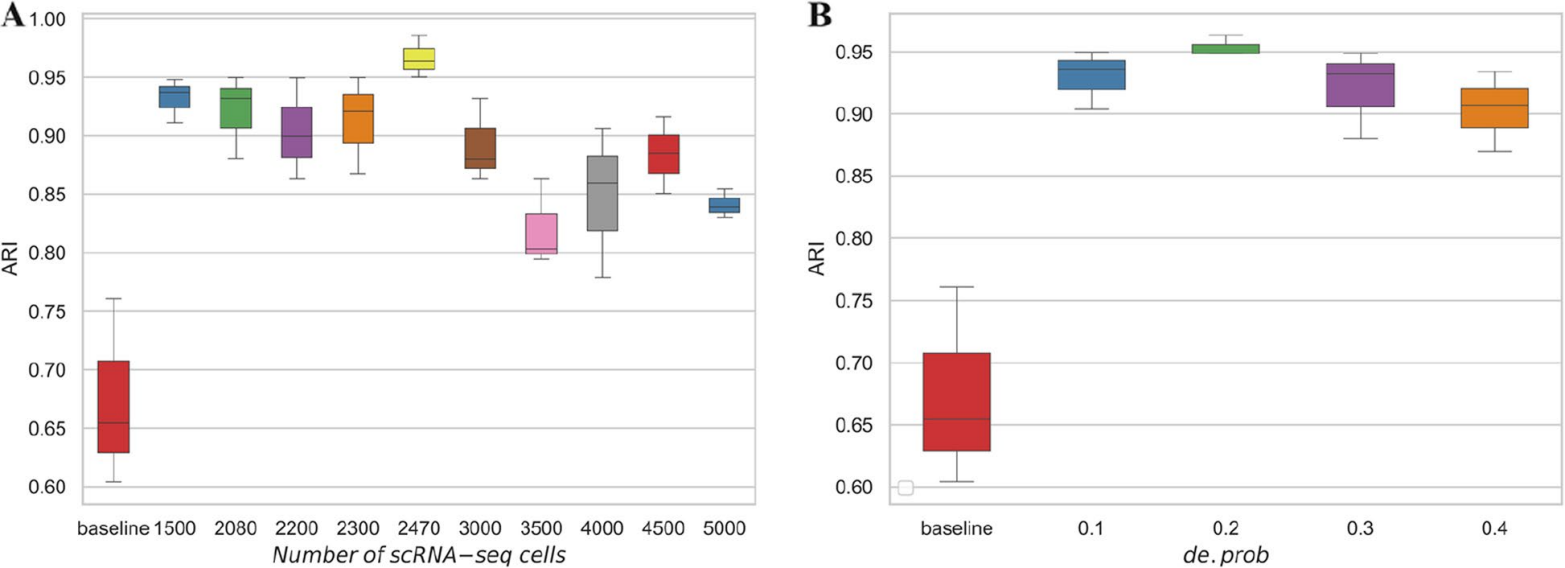
Performance evaluation results of scGAL on simulated datasets. The baseline model only uses the single-cell copy number data to cluster cells. For each configuration of the parameters used for simulating scRNA-seq data, the evaluation is conducted three times on SA501 dataset by using different seeds. **A** Evaluation results on datasets with different numbers of scRNA-seq cells. The results show scGAL performs robustly on different-sized datasets and surpasses the baseline model by a large margin. **B** Evaluation results on datasets generated with different values of the de.prob parameter of Splatter tool. The results showcase scGAL’s high robustness against the distribution changes of scRNA-seq data, and also performs much better than the baseline model

The robustness of scGAL is evaluated by checking if the clustering accuracy changes significantly with respect to the change in data size or distribution. We first compare the clustering results of scGAL on simulated datasets with varying number of scRNA-seq cells. The model that only uses the single-cell copy number data to cluster cells acts as the baseline model. The results in Fig. 9A show the performance of scGAL is robust to data size change and consistently achieves better results than the baseline model, which indicates high effectiveness of our method in learning the representations of scDNA-seq cells, by borrowing complementary information from independent scRNA-seq data. The results also showcase single-cell multi-omics data can provide better characterization of the ITH than individual omics. As shown in Fig. 9B, when evaluated on datasets with varying distributions of gene expression data, our method still achieves high clustering accuracy across different values of the de.prob parameter, and surpasses the baseline model by a large margin, suggesting our method has high robustness against the distributions of scRNA-seq data.

## Discussion and conclusions

Accurately inferring clonal architecture is essential for understanding intra-tumor heterogeneity, and helps us better design personalized treatment strategies. In addition, gene mutations that contribute to tumor metastasis can be identified by comparing clonal architectures inferred from primary and metastatic carcinomas, thus giving clues for the mechanism of tumor metastasis. With a comprehensive characterization of intra-tumor heterogeneity, we can also reveal the lineage relationship between tumor subclones, and gain insights on the evolutionary patterns of tumor.

scDNA-seq provides a convenient way to profile CNAs at single-cell resolution, thereby tumor clones can be identified by clustering the cells. As scDNA-seq data are often complicated by technical factors such as amplification bias and dropouts, single-cell copy numbers inferred from scDNA-seq data are error-prone, which gives rise to a critical challenge of accurately reasoning clonal substructure. Nowadays, single-cell multi-omics data are available for investigating the landscape of cellular heterogeneity from multiple views, and intercorrelations between individual omics data can be exploited to counteract defect of each omics data. Particularly, integrated analysis of single-cell copy number and gene expression data can provide more refined identification of the ITH [31]. In this paper, we introduce scGAL, a hybrid model that combines an AE with a GAN to infer clonal copy number substructure. Our method jointly models independent single-cell copy number and gene expression data from the same cell line under an adversarial learning framework, which relieves the effects of noise in copy number data, and yields semantically meaningful latent representations.

Despite that scGAL is effective in inferring clonal copy number substructure, it does not make a domain alignment between scDNA-seq and scRNA-seq data, and thus cannot jointly cluster the cells from both omics to explicitly build the intercorrelations between copy number and gene expression. Further improvement of scGAL can be achieved by embedding single-cell copy number and gene expression data into a common space, where alignments between scDNA-seq cells and scRNA-seq cells can be conducted by clustering. In addition, attention-based architectures [27] may also be helpful for inferring the ITH from sparse tumor single-cell data, and we plan to explore in this direction in the near future.

## Methods

### Data preprocessing

The proposed scGAL method explores ITH from single-cell copy number data through integrated analysis of independent scRNA-seq data from the same cell line. Due to the high-dimensional and noisy nature of single-cell copy number and gene expression data, directly analyzing original data may yield less accurate results, therefore we employ several preprocessing steps to reduce the dimensionality. For the $N\times M$ single-cell copy number matrix containing copy numbers of $N$ cells in $M$ genomic segments, we perform a log2 transformation of the data and replace resulting NaNs with 0 for computational convenience [20], then reduce the dimensionality following three steps: (1) merge adjacent and identical columns; (2) remove columns with same values in all cells; and (3) select 1024 most informative features based on the variation coefficient. For the single-cell gene expression data, we use the same preprocessing approaches for dimensionality reduction. Furthermore, we divide the UMI count by the total UMI count of each cell to perform normalization of the gene expression matrix, then multiply the data by the median of the total UMI counts of all cells. Finally, we add 1 to the resulting gene-normalized expression matrix as pseudo counts, and perform a log2 transformation of the data [45, 46]. The resulted single-cell copy number and gene expression data are denoted by $X$ and $Y$, respectively.

### Learning low-dimensional representations

The design of scGAL model is mainly inspired by two previous works, one of which jointly analyzes scDNA-seq and scRNA-seq data to provide new insights into maintaining and driving genetic cell diversity in vitro [16], and the other successfully learns latent representations of cells from scRNA-seq data using deep learning models [47]. To obtain informative representations from independent scDNA-seq and scRNA-seq data, scGAL uses an AE to learn latent representations of scDNA-seq cells, and a GAN to emulate corresponding gene expression data from the latent representation of each cell. The joint optimization of the AE and GAN modules enables the model to explore and establish correlations between copy number states and gene expression. This effectively embeds the complementary information from scRNA-seq data into the latent representations of scDNA-seq cells, generating fused semantic features for better inference of clonal substructure. Formally, we utilize the encoder $\phi$ of scGAL to obtain a latent representation $z$ of each $x$ (one row of the $X$), and reconstruct $x$ using a decoder ${\theta }_{1}$, meanwhile another decoder ${\theta }_{2}$ is employed to generate gene expression data $\widehat{y}$ given the $z$. The synthetic $\widehat{y}$ as well as the real gene expression data $y$ (one row of the $Y$) are taken as inputs of the discriminator $D$ to distinguish between real and fake samples. For ease of understanding, we use the generator network $G$ to denote the decoder ${\theta }_{2}$ in the following descriptions. The $G$ and $D$ form a dynamic “game process”, and this process continues until the discriminator $D$ cannot accurately distinguish the real gene expression data from the synthetic data.

To learn the weights of the networks, we train our model by borrowing some ideas from cycle-GAN [48], and use adversarial loss $\mathcal{L}\left(y,z;G,D\right)$ as well as reconstruction loss $\mathcal{L}\left(x,\widehat{x};\phi ,{\theta }_{1}\right)$ together:

1 $$\mathcal{L}\left(x,\widehat{x},y,z;\phi ,{\theta }_{1},G,D\right)=\mathcal{L}\left(y,z;G,D\right)+\lambda \mathcal{L}\left(x,\widehat{x};\phi ,{\theta }_{1}\right)$$

where $\lambda$ is a weight factor to balance the two types of loss functions. The adversarial loss and reconstruction loss are defined as follows:

2 $$\mathcal{L}\left(y,z;G,D\right)={E}_{y\sim {p}_{\text{data}}\left(y\right)}\left[\text{log}D\left(y\right)\right]+{E}_{z\sim{p}_{z}\left(z\right)}\left[\text{log}\left(1-D\left(G\left(z\right)\right)\right)\right]$$

3 $$\mathcal{L}\left(x,\widehat{x};\phi ,{\theta }_{1}\right)=\frac{1}{2 M}{\parallel {x}-\widehat{x}\parallel}_{2}^{2}$$

As our model takes both single-cell copy number and gene expression data as input, we determine their respective batch sizes in each training iteration based on the relative size of each type of data. Subsequently, we feed these two types of omics data into the model and update network parameters by calculating the adversarial loss and reconstruction loss for a batch of samples. Specifically, the discriminator $D$ is trained to accurately classify both real and fake samples by maximizing the probability of corresponding labels, while the generator $G$ is trained to produce samples that can deceive the discriminator $D$ by minimizing $\text{l}\text{o}\text{g}(1-D(G\left(z\right))$. Therefore, $D$ and $G$are engaged in a minimax game through the value function $\mathcal{L}\left(y,z;G,D\right)$. To improve the stability of the model training [49], we follow the strategy introduced in [50] to establish a buffer pool used to store 64 previously generated samples. During training, each newly generated sample has a 50% chance of being used to train the discriminator along with the previously generated samples contained in the buffer pool.

We update model parameters using the Adam optimizer [51]. Adam has been shown to be effective in training deep neural networks as it combines the benefits of the AdaGrad and RMSProp optimization algorithms. Specifically, it dynamically adjusts the learning rate according to the first and second moments of the gradient, leading to faster convergence and better generalization performance. After the model converges, we obtain the latent representation $z$ of each scDNA-seq cell through the encoder network.

### Clustering the cells

Following our previous work on clustering single-cell read counts data [19], we cluster the cells by fitting a set of Gaussian mixture models to the latent representations $Z$ of all scDNA-seq cells. The parameters of the GMMs are estimated using the Expectation-Maximization algorithm. Suppose the number of clusters, i.e. the number of components in the GMM, is denoted by $K$, the optimal value of $K$ is then determined by finding the GMM that has the minimum BIC. Specifically, we set the initial value of $K$ to 1 and iteratively increase the value of $K$ by one each time to check if the BIC decreases, and terminates the process if the minimum BIC has been unchanged more than 10 times. Solutions containing small clusters with less than 3 cells are marked as invalid and excluded. The effectiveness of the adopted GMM-based clustering approach has been demonstrated in previous studies [20, 21].

## Data Availability

Processed sequencing data of SA501, TOV2295R and OV2295R can be downloaded from 10.5281/zenodo.2363826 [31]. Processed data of NCI-N87 can be obtained from https://github.com/XQBai/CCNMF/tree/master/data/NCI_N87 [32].

## Abbreviations

scDNA-seq: Single-cell DNA sequencing
scRNA-seq: single-cell RNA sequencing
CNA: Copy number alteration
ITH: Intra-tumor heterogeneity
VAE: Variational autoencoder
GAN: Generative adversarial network
GMM: Gaussian mixture model

## References

[1] Nowell PC (1976). The clonal evolution of tumor cell populations. Science.

[2] Schwartz R, Schaffer AA (2017). The evolution of tumour phylogenetics: principles and practice. Nat Rev Genet.

[3] Lawson DA, Kessenbrock K, Davis RT, Pervolarakis N, Werb Z (2018). Tumour heterogeneity and metastasis at single-cell resolution. Nat Cell Biol.

[4] Michor F, Polyak K (2010). The origins and implications of intratumor heterogeneity. Cancer Prev Res (Phila).

[5] de Bruin EC, McGranahan N, Mitter R, Salm M, Wedge DC, Yates L, Jamal-Hanjani M, Shafi S, Murugaesu N, Rowan AJ (2014). Spatial and temporal diversity in genomic instability processes defines lung cancer evolution. Science.

[6] Morris LG, Riaz N, Desrichard A, Senbabaoglu Y, Hakimi AA, Makarov V, Reis-Filho JS, Chan TA (2016). Pan-cancer analysis of intratumor heterogeneity as a prognostic determinant of survival. Oncotarget.

[7] Greaves M (2015). Evolutionary determinants of cancer. Cancer Discov.

[8] Turajlic S, Sottoriva A, Graham T, Swanton C (2019). Resolving genetic heterogeneity in cancer. Nat Rev Genet.

[9] Siravegna G, Mussolin B, Buscarino M, Corti G, Cassingena A, Crisafulli G, Ponzetti A, Cremolini C, Amatu A, Lauricella C (2015). Clonal evolution and resistance to EGFR blockade in the blood of colorectal cancer patients. Nat Med.

[10] McGranahan N, Swanton C (2017). Clonal heterogeneity and tumor evolution: past, present, and the future. Cell.

[11] Gohil SH, Iorgulescu JB, Braun DA, Keskin DB, Livak KJ (2021). Applying high-dimensional single-cell technologies to the analysis of cancer immunotherapy. Nat Rev Clin Oncol.

[12] Kuipers J, Jahn K, Beerenwinkel N (2017). Advances in understanding tumour evolution through single-cell sequencing. Biochim Biophys Acta Rev Cancer.

[13] Zahn H, Steif A, Laks E, Eirew P, VanInsberghe M, Shah SP, Aparicio S, Hansen CL (2017). Scalable whole-genome single-cell library preparation without preamplification. Nat Methods.

[14] Zheng GX, Terry JM, Belgrader P, Ryvkin P, Bent ZW, Wilson R, Ziraldo SB, Wheeler TD, McDermott GP, Zhu J (2017). Massively parallel digital transcriptional profiling of single cells. Nat Commun.

[15] Zhu C, Preissl S, Ren B (2020). Single-cell multimodal omics: the power of many. Nat Methods.

[16] Andor N, Lau BT, Catalanotti C, Sathe A, Kubit M, Chen J, Blaj C, Cherry A, Bangs CD, Grimes SM (2020). Joint single cell DNA-seq and RNA-seq of gastric cancer cell lines reveals rules of in vitro evolution. NAR Genom Bioinform.

[17] Zong C, Lu S, Chapman AR, Xie XS (2012). Genome-wide detection of single-nucleotide and copy-number variations of a single human cell. Science.

[18] Zaccaria S, Raphael BJ (2021). Characterizing allele- and haplotype-specific copy numbers in single cells with CHISEL. Nat Biotechnol.

[19] Yu Z, Liu F, Shi F, Du F (2023). rcCAE: a convolutional autoencoder method for detecting intra-tumor heterogeneity and single-cell copy number alterations. Brief Bioinform.

[20] Rashid S, Shah S, Bar-Joseph Z, Pandya R (2021). Dhaka: variational autoencoder for unmasking tumor heterogeneity from single cell genomic data. Bioinformatics.

[21] Yan J, Ma M, Yu Z (2023). bmVAE: a variational autoencoder method for clustering single-cell mutation data. Bioinformatics.

[22] Gan Y, Huang X, Zou G, Zhou S, Guan J (2022). Deep structural clustering for single-cell RNA-seq data jointly through autoencoder and graph neural network. Brief Bioinform.

[23] Wang J, Xia J, Wang H, Su Y, Zheng CH (2023). scDCCA: deep contrastive clustering for single-cell RNA-seq data based on auto-encoder network. Brief Bioinform.

[24] Zeng P, Wangwu J, Lin Z. Coupled co-clustering-based unsupervised transfer learning for the integrative analysis of single-cell genomic data. Brief Bioinform. 2021;22(4):bbaa347. 10.1093/bib/bbaa347 33279962

[25] Zou G, Lin Y, Han T, Ou-Yang L. DEMOC: a deep embedded multi-omics learning approach for clustering single-cell CITE-seq data. Brief Bioinform. 2022;23(5):bbac347. 10.1093/bib/bbac347 36047285

[26] Eltager M, Abdelaal T, Mahfouz A, Reinders MJT (2022). scMoC: single-cell multi-omics clustering. Bioinform Adv.

[27] Ren L, Wang J, Li Z, Li Q, Yu G (2023). scMCs: a framework for single-cell multi-omics data integration and multiple clusterings. Bioinformatics.

[28] Ye X, Shang Y, Shi T, Zhang W, Sakurai T (2023). Multi-omics clustering for cancer subtyping based on latent subspace learning. Comput Biol Med.

[29] Rong Z, Liu Z, Song J, Cao L, Yu Y, Qiu M, Hou Y (2022). MCluster-VAEs: an end-to-end variational deep learning-based clustering method for subtype discovery using multi-omics data. Comput Biol Med.

[30] Ge S, Liu J, Cheng Y, Meng X, Wang X (2023). Multi-view spectral clustering with latent representation learning for applications on multi-omics cancer subtyping. Brief Bioinform.

[31] Campbell KR, Steif A, Laks E, Zahn H, Lai D, McPherson A, Farahani H, Kabeer F, O’Flanagan C, Biele J (2019). Clonealign: statistical integration of independent single-cell RNA and DNA sequencing data from human cancers. Genome Biol.

[32] Bai X, Duren Z, Wan L, Xia LC. Joint Inference of Clonal Structure using Single-cell Genome and Transcriptome Sequencing Data. bioRxiv. 2020.02.04.934455. 10.1101/2020.02.04.934455. 10.1093/nargab/lqae017 PMC10939367 38486887

[33] Edrisi M, Huang X, Ogilvie HA, Nakhleh L (2023). MaCroDNA: accurate integration of single-cell DNA and RNA data for a deeper understanding of tumor heterogeneity. bioRxiv.

[34] Zafar H, Navin N, Chen K, Nakhleh L (2019). SiCloneFit: bayesian inference of population structure, genotype, and phylogeny of tumor clones from single-cell genome sequencing data. Genome Res.

[35] He K, Zhang X, Ren S, Sun J. Delving Deep into rectifiers: Surpassing Human-Level performance on ImageNet classification. In.; 2015: arXiv:1502.01852.

[36] Chen Z, Gong F, Wan L, Ma L (2020). RobustClone: a robust PCA method for tumor clone and evolution inference from single-cell sequencing data. Bioinformatics.

[37] Satija R, Farrell JA, Gennert D, Schier AF, Regev A (2015). Spatial reconstruction of single-cell gene expression data. Nat Biotechnol.

[38] Wang Y, Yu Z, Li S, Bian C, Liang Y, Wong KC, Li X (2023). scBGEDA: deep single-cell clustering analysis via a dual denoising autoencoder with bipartite graph ensemble clustering. Bioinformatics.

[39] Yu ZH, Lu YF, Wang YH, Tang F, Wong KC, Li XT (2022). ZINB-based graph embedding autoencoder for single-cell RNA-Seq interpretations. Aaai Conf Artif Inte.

[40] Meilă M (2007). Comparing clusterings—an information based distance. J Multivar Anal.

[41] Knops ZF, Maintz JB, Viergever MA, Pluim JP (2006). Normalized mutual information based registration using k-means clustering and shading correction. Med Image Anal.

[42] Rousseeuw P (1987). Silhouettes: a graphical aid to the interpretation and validation of cluster analysis. J Comput Appl Math.

[43] Calinski T, Harabasz J (1974). Simulation JJCiS, Comp: A Dendrite Method for Cluster Analysis.

[44] Zappia L, Phipson B, Oshlack A (2017). Splatter: simulation of single-cell RNA sequencing data. Genome Biol.

[45] Luecken MD, Theis FJ (2019). Current best practices in single-cell RNA-seq analysis: a tutorial. Mol Syst Biol.

[46] Sinha D, Kumar A, Kumar H, Bandyopadhyay S, Sengupta D (2018). dropClust: efficient clustering of ultra-large scRNA-seq data. Nucleic Acids Res.

[47] Gronbech CH, Vording MF, Timshel PN, Sonderby CK, Pers TH, Winther O (2020). scVAE: variational auto-encoders for single-cell gene expression data. Bioinformatics.

[48] Zhu JY, Park T, Isola P, Efros AA. Unpaired Image-to-Image Translation Using Cycle-Consistent Adversarial Networks. 2017 IEEE International Conference on Computer Vision (ICCV). Venice; 2017. p. 2242-51. 10.1109/ICCV.2017.244.

[49] Goodfellow IJ, Pouget-Abadie J, Mirza M, Xu B, Warde-Farley D, Ozair S, Courville A, Bengio Y. Generative adversarial nets. In Proceedings of the 27th International Conference on Neural Information Processing Systems - Volume 2 (NIPS 2014). Cambridge: MIT Press; 2014. p. 2672–80.

[50] Shrivastava A, Pfister T, Tuzel O, Susskind J, Wang W. Webb RJae-p: learning from simulated and unsupervised images through adversarial training. 2016: arXiv:1612.07828.

[51] Kingma DP, Ba J. Adam: A Method for Stochastic Optimization. 3rd International Conference on Learning Representations (ICLR 2015). San Diego; 2015. 10.48550/arXiv.1412.6980.

